# 

**Published:** 1918-04-06

**Authors:** 


### CONTENTS.

PAGE

**The Crucifixion of Love** ... ... ... ... ... 1

**Fitness for Military Service** ... ... ... 2

**Hospital and Institutional News** ... ... ... 3

Curative Work in the Eastern Command—The Million Half-crowns—A Group of Nerve Hospitals for Pensioners—The Promotion of Major Rhodes—Civilian Beds and the Great Offensive—A New Departure at the Cancer Hospital—The Robert Burns Cots: A Suggestion—A Hospital Secretary's Legacy—A War Memorial at Tunbridge Wells—A Battalion's Remarkable Gift—The Fruits of Good Work at Chesterfield—The Penalty of Inexperience—The Patients of St. Dunstan's—The Danger of the Unvaccinated—New Scheme for Social Workers—Nurses and the Meat Ration—Co-ordination in Treatment of Limbless Cases—A New *Gazette*—This Week's Drug Market.

**The Great War** ... ... ... ... ... ... 7

V. Sickness.

**The Abuse of Salt and Vinegar** ... ... ... 8

By G. Arbour Stephens, M.D.

**Lord Lister** ... ... ... ... ... ... ... 9

XI. London's Indifference to Listerism.

**Recent Official Publications**... ... ... ... 10

**The Institutional Library** ... ... ... ... 11

Three Clinical Studies in Tuberculous Predisposition—Memoranda on Army General Hospital Administration—The Cause and Prevention of Cancer—The Nation's Health—A Text-Book of War Nursing—Dainty Dishes for Camp and Home.

PAGE

**The American Red Cross** ... ... ... ... 13

The Pioneers of America's United Effort.

**Nursing Affairs in Ireland** ... ... ... ... 14

Propaganda: The Effort Towards Union.

**The Matrons' and Sisters' Department** ... 15

The Growth of the Nursing Profession: VI. Opportunities of Study for the Graduate Nurse.

Round the Hospitals.

The Message of Easter—Nurses' Memorial Service: Latest Arrangements—War-work Depôts after the War—College Local Centres—Manchester's Post Graduate Course—Nurses and Food Production.

**The Editor's Letter-Box** ... ... ... ... 19

Beckett Hospital, Barnsley—Reports of Scottish Asylums.

**Parliament and the Profession** ... ... ... 19

**Some Annual Meetings** ... ... ... ... 20

**The Book Market** ... ... ... ... ... iv

**Annual Meetings** ... ... ... ... ... iv

**Passing Events and Topics** ... ... ... ... iv

**Matrons' and Sisters' Appointments** ... ... iv

**Soldiers and Infected Women** ... ... ... iv

New Defence of the Realm Order.

**The Institutional Worker** ... ... (Suppl.) 1

Hot Dinner-plates: Answer to February Question.
April Question.
Questions Invited.
Enquiries and Answers.
The Sick and in Need.
Employment and Training.
War Matters.
The Housekeepers' Department.

